# Maternal ‘near miss’ collection at an Australian tertiary maternity hospital

**DOI:** 10.1186/s12884-018-1862-6

**Published:** 2018-06-11

**Authors:** Skandarupan Jayaratnam, Sonia Kua, Caroline deCosta, Richard Franklin

**Affiliations:** 1 College of Medicine and Dentistry, James Cook University, Cairns, QLD Australia; 2 King Edward Memorial Hospital, Perth, WA Australia; 3 College of Medicine and Dentistry, James Cook University, Cairns, QLD Australia; 4 College of Public Health, Medical & Veterinary Sciences, James Cook University, Townsville, QLD Australia

**Keywords:** Near miss healthcare, Severe maternal morbidity, Pregnancy complications, Maternal health, Maternal mortality

## Abstract

**Background:**

Australia has a maternal mortality ratio of 6.8/100000 live births, a rate akin to other developed countries and consistent with the high level care provided within the Australian health care system. With maternal mortality at very low levels assessment of severe maternal morbidity is increasingly being used as an indicator of quality of care and to identify areas for improvement in maternity services. The WHO maternal ‘near miss’ criteria is a standardised tool has been increasingly used worldwide to assess maternal morbidity and standards of maternity care. The aim of this study was to determine the rate and aetiology of maternal ‘near misses’ at King Edward Memorial Hospital (KEMH) using the WHO near miss criteria.

**Methods:**

Cases of maternal ‘near miss’ were prospectively identified at KEMH using the WHO near miss criteria over a period of 6 months (1st December 2014 to 31st May 2015). A descriptive analysis of the results was undertaken.

**Results:**

During the study there were 2773 live births with 19 women who had ‘near miss’ presentations. There were no maternal deaths. The maternal ‘near miss’ index rate was 7/1000 live births. The main causes of obstetric ‘near miss’ were obstetric haemorrhage, pre-eclampsia and early pregnancy complications.

**Conclusion:**

The rate of maternal ‘near miss’ at KEMH was 7/1000 live births and post-partum haemorrhage was identified as the most common aetiology, consistent with other studies in developed countries. Further research comparing currently utilised local, state and national morbidity systems would allow further validation of the WHO near miss criteria in Australian settings.

The study presented in this publication was undertaken at King Edward Memorial Hospital, 374 Bagot Rd., Subiaco WA 6008.

**Electronic supplementary material:**

The online version of this article (10.1186/s12884-018-1862-6) contains supplementary material, which is available to authorized users.

## Background

Maternal mortality in Australia has decreased significantly over the last century, from 41.2 per 100,000 women giving birth in 1964–1966 period, to 7.1 per 100,000 women in the years 2008–2012 [1, 2]. This marked improvement has been attributed to many factors including antibiotics and blood transfusion facilities, increased education and socioeconomic prosperity of women, and improvements in the provision of health care [2].

One of the mainstays of addressing deficiencies of maternal care in the health system has been a maternal death audit both at the level of the facility and also nationally through confidential enquiries of maternal deaths [3]. These have allowed the assessment of each maternal death to determine aetiology and the factors to be addressed to ensure improved provision of care. However, as maternal mortality rates have decreased in developed countries, maternal deaths especially at a single institution have become rare. Furthermore, the deaths are often more medically complicated and the information gained from reviewing these deaths for broader obstetric practice, may not be as pertinent. In this low mortality setting, the concept of monitoring ‘near miss’ events or severe maternal morbidity has been introduced as a means of attaining valuable information on the quality of obstetric care [4].

Maternal near miss and maternal deaths share many characteristics and pathological processes [4]. Moreover there are advantages in the assessment of near miss cases in that they are more frequent relative to maternal deaths and provide greater capacity to understand health system limitations, while still being infrequent enough not to overload clinical audit capacity [4, 5]. Additionally, there is the capacity to converse with the patient and family following recovery to understand issues that may have contributed to the poor outcomes.

**Fig. 1:**
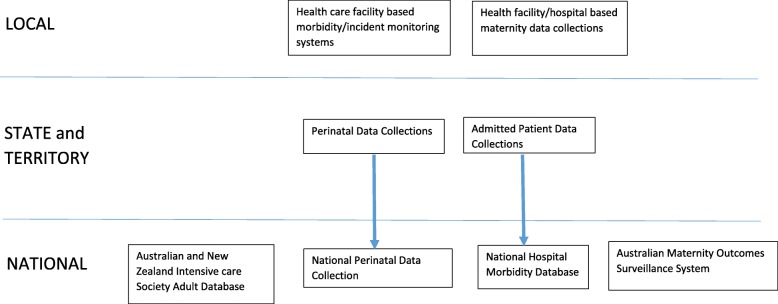
Sources of Severe Maternal Morbidity Information in Australia. Adapted from Figure 1-Relationships between data collections included in the review in Maternity data in Australia: a review of sources and gaps. AIHW National Perinatal Epidemiology and Statistics Unit. Bulletin 87. September 2011

In 2009 the WHO published near miss criteria to provide a standardised approach to identify near miss in both individual institutions and larger health care systems [5]. The subsequent analysis of the health care system would thus allow for the development of interventions to improve maternity health care [4]. While there has been increased uptake of the WHO near miss criteria in developing nations, most developed nations have continued to utilise their own local, state or national morbidity reporting systems. There has been limited utilisation of WHO ‘near miss’ criteria in the Australian context, with only two studies undertaken, both in regional centres [6, 7]. They do however highlight the utility of the systematic collection of cases of severe morbidity. Information regarding maternal morbidity in Australia can be attained through a number of differing data collection systems (Fig. 1). Unlike maternal deaths, where notification in all jurisdictions leads to information being centralised through the National Maternal Deaths Database (NMDD), there is currently no central database for severe maternal morbidity in Australia [8]. Most maternal morbidity data is extracted from state and territory perinatal data collections and through administrative data collections [8, 9]. Information about severe maternal morbidity may also be collected using local hospital incident systems. These systems are designed to identify and investigate a variety of critical clinical incidents within the hospital system, and are not necessarily specific to the purpose of identifying severe obstetric morbidity [7], though many of them are incorporated in the collection systems.

The aim of this study was the application of the WHO near miss criteria and to assess its utility in identifying cases of severe maternal morbidity in a developed tertiary hospital setting.

## Methods

### Setting

King Edward Memorial Hospital for Women (KEMH), Western Australia’s largest maternity hospital, is located in Perth, Western Australia (WA). It provides pregnancy and neonatal care within the greater Perth Metropolitan area and is the main referral centre for complex, tertiary maternity care for the state population of 2.6 million people [10], which includes rural and remote communities. The maternity unit is staffed by 21 obstetric staff specialists and supported by an Obstetric High Dependency Unit (HDU) with 6 beds, and an 80 bed Neonatal Intensive Care Unit (NICU). Any cases requiring more intensive care treatment or radiological intervention (e.g. uterine artery embolisation) are transferred to the Intensive Care Unit (ICU) at Sir Charles Gardiner Hospital (SCGH) located 3 km away.

Among the clinical audit sytems in use at KEMH is the Clinical Incidence Monitoring System (CIMS), a hospital database incorporating reporting of clinical cases where adverse maternal outcomes or deviations of care from standard protocol occur. CIMS reporting is strongly encouraged but is not mandatory. Any health professional can place a clinical incident into the system, and is then presented to the appropriate clinical incident review expert panel committee at KEMH Some of these cases relate to severe life threatening scenarios, but also include less life threatening morbidity such as 4th degree tears or deviations from standard practice of care e.g. caesarean section for a second twin.

### Inclusion criteria and definitions

This prospective observational study included all maternal ‘near misses’ and deaths at KEMH between 1st Dec 2014 and 31 May 2015. During this period, all women admitted to KEMH during pregnancy or within 42 days of its termination were eligible for inclusion. A form consisting of clinical and biochemical parameters indicative of severe obstetric morbidity was broadened to include specific disease entities and interventions, thus allowing easy identification of potential ‘near miss’ cases (Additional file 1). These cases were then further reviewed by the authors to determine which cases fulfilled the WHO criteria for ‘near miss’ (Additional file 2) to warrant study inclusion. Cases of maternal ‘near miss’ were classified by the primary underlying cause defined as the disease process most likely leading to the ‘near miss’.

### Data collection and analysis

Cases were identified and collected on a daily basis by the authors, through participation in unit meetings and regular visits to the labour ward, in-patient obstetric and gynaecology wards and Obstetric HDU. The paper and electronic records of each potential ‘near miss’ case were reviewed. When one of the clinical, laboratory or management criteria were met, a structured data form was completed, including age, parity, primary residence during the pregnancy and duration of hospital stay and incorporated onto an Excel spread sheet. Potential ‘near miss’ cases were independently reviewed by two of the investigators to determine if they met WHO criteria for ‘near miss’ and to identify the primary underlying cause. A descriptive analysis of the results was conducted following completion of the audit. The Maternal Near Miss ratio (MNMR) was calculated by the number of maternal near-miss cases per 1000 live births [11]. Primary residence during pregnancy was categorised according to the Australian Institute of Health and Welfare criteria as either Metropolitan (Capital city or other urban centre with population > 100,000), rural (population < 100,000) or remote (population < 5000). For the purposes of this study rural and remote were grouped together under ‘Rural’ [12].

## Results

During the 6-month study period there were 2773 live births: 19 cases of near miss and no maternal deaths. The MNMR was 7/1000. Three cases required transfer to Intensive Care Unit of a nearby general tertiary hospital while all the rest were managed within the setting of the High Dependency Unit (HDU) at KEMH.

Most cases of severe maternal outcome were from direct causes with the most common aetiology being postpartum haemorrhage, pre-eclampsia and early pregnancy complications (Table 1). Indirect aetiology was present in four cases.

**Table 1:** Aetiology of Maternal ‘Near miss’ at KEMH

Gestational Age	Maternal Near Miss	WHO near miss criteria fulfilled
< 12 weeks (1st trimester)	Ruptured cornual ectopic pregnancy	Transfusion of > = 5 U blood
	Ruptured tubal ectopic pregnancy	Transfusion of > = 5 U blood
	Septic miscarriage	Intubated and ventilated
	Complication of miscarriage	Required hysterectomy and transfusion of >=5 U of blood
13–27 weeks	Complicated second trimester miscarriage	Transfusion of > = 5 U blood
	HELLP	Platelets< 50
28-40 weeks (3rd trimester)	Pre-eclampsia & Pancreatitis	Needing intubation and ventilation and altered conscious state
	HELLP	Platelets< 50
	Massive PPH	Peripartum hysterectomy and blood transfusion >= 5 U
	Placenta increta	Peri-partum hysterectomy
	Placenta increta and PPH	Peri-partum hysterectomy and blood transfusion of >= 5 U
	PPH and early DIC	Transfusion of > = 5 U blood
	Diabetic ketoacidosis	Ph < 7.1, RR < 6, intubated and ventilated
	Acute Respiratory Distress Syndrome (ARDS)	Intubated and ventilated
	Anaesthetic complication post regional anaesthesia	Intubated and ventilated
	Malaria (Falciparum)	Platelet < 50 (33)
	HELLP	Platelet < 50 (44)
	Acute Fatty Liver of Pregnancy	PO2 < 90
	PPH	Intubated & ventilated

The mean age of the near miss case was 29, while the median parity was 1. The majority (14 women) were residing in metropolitan areas while 5 women came from rural or remote locations. The mean age of the background cohort was 30, while the median parity was 1. 14.6% of women admitted to KEMH were from rural and regional areas during the 2014/2015 year.

Near miss cases were more common in the third trimester with 67% of cases. Complications during the first and second trimester accounted for 22 and 11% of cases respectively.

## Discussion

The ‘near miss’ rate of 7 per 1000 live-births was consistent with other studies on severe maternal morbidity in Australia and in developed countries internationally [6, 7, 13–18]. Australian studies using routinely collected data reveal a similar figure of maternal morbidity in Victoria and NSW [19, 20].

Maternal near miss: mortality ratio (MNM:MR) and MMR were not able to be calculated for this cohort as no deaths were present in the study. The MNM:MR tends to reflects quality of care within the health facility with a low ratio indicating poorer care with large number of near miss cases progressing onto maternal deaths. Near miss studies undertaken in developing countries in the region have highlighted this, though consideration must also be given to the late presentation of such patients where limited care can only be provided to those in such moribund states. The last death at KEMH was more than 7 years ago, reflecting the rarity of maternal mortality in an individual obstetric unit in a well-resourced setting.

Unsurprisingly, all cases of severe Obstetric morbidity received critical care support consistent with the capacity of tertiary maternity hospitals in well-resourced settings. Studies in Cairns and Darwin also showed high levels of critical care admission/support well above the 70% recommended by the WHO [6, 7, 11].

This audit found that post-partum haemorrhage (PPH), pre-eclampsia and early pregnancy complications (ectopic pregnancies and miscarriage) were common contributors to severe morbidity, consistent with national and international surveillance reports [17, 18, 21, 22].Although death from PPH in Australia is rare, there has been a rise in PPH incidence in Australia and subsequent increased rate in severe adverse outcomes [23]. The incidence of pre-eclampsia in Australia has reduced over the last decade but morbidity particularly eclampsia has not substantially decreased [24]. These findings reinforce the continued need to be ever vigilant even in well-resourced settings to ensure that lessons learned from reduction in maternal deaths continue to be pertinent in the modern management of severe maternal morbidity.

There were many clinical incidents reported during the 6-month study but none of the near misses were Severity Assessment Code 1 (SAC1) cases, which reflect the most severe cases systemic issues leading to adverse outcomes. Our study indicated the CIMS is not geared to singularly identify maternal near miss or severe obstetric morbidity and relies on accurate reporting by staff members which is a significant limitation of voluntary databases. A recent near miss study in Darwin [7] showed similar findings with over 267 incidents reported and only 10 near misses identified during the audit period [7]. The differences obtained illustrate the different criteria used to define severe obstetric morbidity and the incorporation of many cases not related to severe obstetric morbidity into hospital audit systems. These examples highlight the need for a specific database related to severe obstetric morbidity as well as persistent and accurate surveillance within hospitals to clearly identify and study relevant cases. Research using state and territory-based data sets has allowed identification of severe morbidity [19, 20] though difficulties exist in ensuring all cases are recognized. To address the existence of differing systems measuring morbidity data, data linkage has been proposed to incorporate various parameters in the development of a composite score indicating the level of severe obstetric morbidity [25]. This is a positive step in the identification and measurement of morbidity in Australia, though further consensus on the criteria defining severe obstetric morbidity is required for improved utilisation of such tools.

The WHO criteria classified five of the cases not fulfilling CIMS criteria as true near misses. The cases were related to criteria of decreased platelets (< 50), use of vasoactive agents and increased respiratory rate. Acute thrombocytopenia is one of the more common laboratory findings meeting criteria for WHO near miss [26] and as noted in the use of maternal severity index (MSI) scoring system [27] decreased platelets by themselves do not portend a significant risk of progressing to death. This is in contrast to criteria such as cardiac arrest and the need for intubation and ventilation, which bode a much worse outcome. Increased respiratory rate in the context of severe morbidity has a high mortality index; however, this may be more relevant to persistent respiratory compromise rather than transient respiratory distress in a condition such as pneumonia.

This raises the question of whether certain criteria e.g. increased RR and decreased platelets should be incorporated as individual entities in the maternal near miss criterion or rather be utilised as an adjunct to other WHO near miss criteria. Additionally, six cases were included based on the transfusion of more than 5 units of blood. The availability of blood is variable between different settings but there are also differing thresholds among clinicians in the provision of blood transfusion [28]. Therefore the usage of blood transfusion ≥ 5 U as criteria may be an indicator of access rather than true reflection of severity. Further work is required to understand the utilisation of blood transfusions as an indicator of obstetric severity. In our context, the threshold for intubation and ventilation is also variable and may not necessarily reflect severity but rather the threshold for intervention of each individual practitioner. This is particularly important for patients transferred from rural and regional areas who are under the care of GP anaesthetists, with relatively fewer resources to provide ongoing management of high acuity patients.

### Limitations of the study

There was a significant effort in attaining cases of potential near miss and then reviewing those cases to identify true WHO near miss criteria. Regular diligent review of clinical areas by the authors and supporting staff was required and as such it is possible, but unlikely that there were cases that were missed. However, it must be emphasised that such a prospective collection requires a significant input of manpower and time. It is hoped that as the WHO ‘near miss’ tool develops and is adapted into practice, software may be developed to automatically identify near misses using routinely collected parameters minimising the error of missing cases.

The short audit time also limited the number of cases attained during the study. A longer study period would provide a more accurate trend of near miss at KEMH and allow more robust comparison of the WHO near miss tool to currently utilised morbidity systems in Australia. In particular comparisons of the WHO near miss system to validated tools such as Maternal Morbidity Outcome Indicator (MMOI) [25] developed from routinely collected data would help in identifying aspects of the WHO near miss criteria which may be incorporated into the development of morbidity tools.

A longer audit period would also have facilitated epidemiological analysis of cases with attention to identifying risk factors e.g. age, parity and areas for clinical improvement within the health facility. Our study showed a relatively low parity among our cases with 5 women from rural/regional areas though any meaningful analysis would require larger number of clinical cases. Studies have indicated that age, SES status and rurality among other factors as significant risk factors for morbidity and a larger cohort would allow identification of whether these are apparent [19, 20].

Though review of clinical management of near miss cases was outside the scope of this study, the study of near miss cases provides a unique opportunity to not only objective access institutional care but to understand the circumstances which may have led to the near miss from the perspective of recovered patients and their families.. An in-depth review of a larger cohort of cases may show avenues for clinical improvement and a greater comprehension of issues important to patients experiencing near misses.

## Conclusion

The WHO near miss rate of 7/1000 at KEMH is consistent with studies in other similar developed economy settings. The WHO near miss criteria was able to identify cases of severe morbidity and may provide a suitable framework for determining cases of maternal near miss in an Australian setting. However, further refinement of the WHO criteria to the Australian context along with comparisons to other morbidity collections systems in Australia is required.

## Additional files

Additional file 1: King Edward Memorial Hospital Maternal ‘Near Miss’ collection form. Data collection tool for the collection of maternal near miss cases at KEMH. (DOCX 19 kb).

Additional file 2: WHO near miss criteria. WHO near miss criteria is illustrated and divided into 3 main categories: clinical, laboratory and management based criteria. (DOCX 85 kb).

## Abbreviations

HDU: High Dependency Unit
HeLLP: Haemolysis, Liver enzymes, Low Platelets
ICU: Intensive care unit
KEMH: King-Edward Memorial Hospital
MMR: Maternal Mortality ratio
MNM:MR: Maternal near miss: mortality ratio
MNMR: Maternal near miss ratio
MSI: Maternal Severity Index
PPH: Postpartum haemorrhage
SMO: Severe maternal outcomes
WHO: World Health Organisation
