# Supplementary material for: Maternal ‘near miss’ collection at an Australian tertiary maternity hospital
Source: BMC Pregnancy Childbirth. 2018 Jun 11;18:221. doi: 10.1186/s12884-018-1862-6 (PMC5996518; doi:10.1186/s12884-018-1862-6)
Supplement: Supplementary file 1 — King Edward Memorial Hospital Maternal ‘Near Miss’ collection form. Data collection tool for the collection of maternal near miss cases at KEMH. (DOCX 19 kb). [file 12884_2018_1862_MOESM1_ESM.docx]

**Additional file 1**

**King Edward Memorial Hospital Maternal 'Near Miss' collection form**

***FOR ANY PATIENT PRESENTING TO KEMH who fulfils the criteria for a potential near miss or severe obstetric morbidity below, please fill out this form and place in the 'Near Miss' collection box (Doctors Common room) or give in person to DR S.JAYARATNAM OR DR Sonia KUA***

**PATIENT STICKER**

- Type of potential 'near miss' (please tick appropriate box below):

|  | Any antepartum haemorrhage (APH) transferred as an emergency for **maternal reasons** to operating theatre **(OT)** e.g. Massive APH with maternal compromise |
| --- | --- |
|  | Any PPH transferred to OT requiring **Laparotomy e.g.** requiring B-Lynch or peri-partum hysterectomy (please include elective peri-partum hysterectomy for invasive placental disease e.g. accrete)) |
|  | Any Bleed (APH/PPH/ruptured ectopic/miscarriage) requiring **>=5U blood transfusion** |
|  | Any patient in the **POSTNATAL WARDs transferred to OT/ASCU/LBS** e.g. eclamptic fit on the ward or haemoperitoneum post delivery/operation requiring transfer to OT |
|  | **Severe pre-eclampsia** complicated by HELLP syndrome, eclampsia, acute renal failure (creatinine >300), DIC or pulmonary oedema or other major morbidity |
|  | Any patient transferred to **ICU** i.e. transferred out of KEMH |
|  | Any ruptured ectopic pregnancy requiring **Laparotomy** |
|  | Any patient experiencing **severe shock** or **unexplained maternal collapse** |
|  | Any **pulmonary embolus** |
|  | Any **other conditions** which required **immediate medical or anaesthetic assessment** e.g. Respiratory/cardiac compromise |
