# Supplementary material for: Maternal ‘near miss’ collection at an Australian tertiary maternity hospital
Source: BMC Pregnancy Childbirth. 2018 Jun 11;18:221. doi: 10.1186/s12884-018-1862-6 (PMC5996518; doi:10.1186/s12884-018-1862-6)

**Additional file 2** **– WHO near miss criteria**: a woman presenting with any of the following life-threatening conditions and surviving a complication that occurred during pregnancy, childbirth or within 42 days of termination of pregnancy should be considered as a maternal near miss case^5^. Note the 3 different criteria (clinical, laboratory and management based criteria) used to identify cases of ‘near miss’.


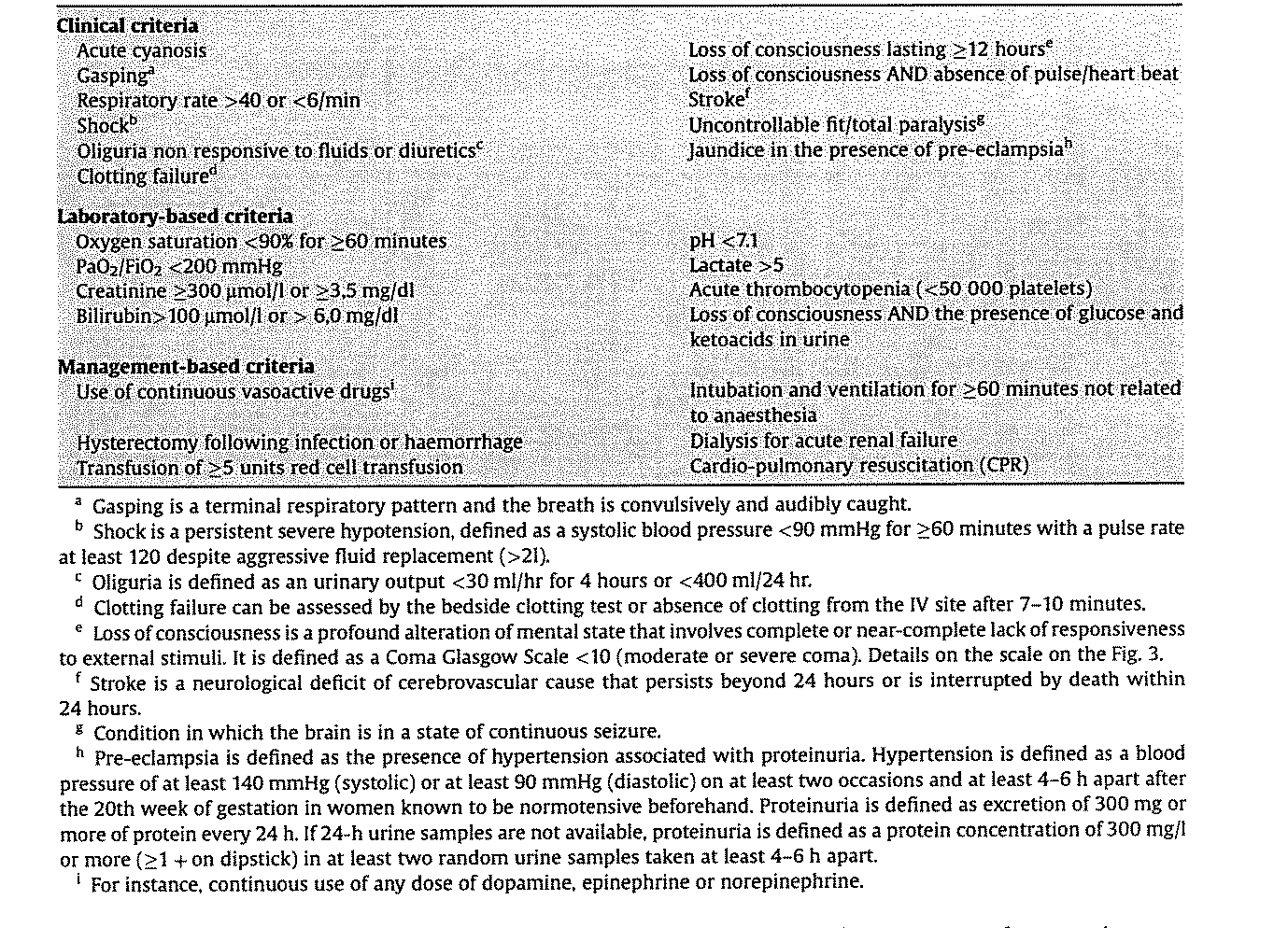

Supplement: Supplementary file 2 — WHO near miss criteria. WHO near miss criteria is illustrated and divided into 3 main categories: clinical, laboratory and management based criteria. (DOCX 85 kb). [file 12884_2018_1862_MOESM2_ESM.docx]
